# Post-operative complications following cervical ventral slot in dogs: a retrospective review of the influence of prophylactic fenestration and chondrodystrophy in 593 cases

**DOI:** 10.3389/fvets.2025.1616461

**Published:** 2025-07-29

**Authors:** Christophe Osterreicher Cunha Dupont, Giunio Bruto Cherubini

**Affiliations:** ^1^Dick White Referrals, Linnaeus Veterinary Limited, Cambridgeshire, United Kingdom; ^2^Department of Veterinary Science, Veterinary Teaching Hospital “Mario Modenato”, University of Pisa, Pisa, Italy

**Keywords:** prophylactic fenestration, intervertebral disc herniation, cervical, dog, canine, complications, chondrodystrophy

## Abstract

**Introduction:**

Cervical intervertebral disc herniation is a significant health concern in dogs, leading to pain, neurological deficits, and a high rate of recurrence that can compromise both quality of life and long-term mobility. Prophylactic fenestration has demonstrated benefits in reducing recurrence in the thoracolumbar spine. Although its application in the cervical spine remains debated, it is routinely performed in some practices with the aim of preventing future disc herniations. However, no published data have previously addressed the safety or perioperative risks of cervical prophylactic fenestration.

**Objective:**

To describe the frequency, distribution, and clinical associations of prophylactic fenestration performed during cervical decompressive surgery, and to assess its relationship with perioperative complications.

**Materials and methods:**

A retrospective review of clinical records from a single referral institution (2010–2020) was conducted of dogs undergoing cervical ventral slot surgery for intervertebral disc extrusion. Associations between prophylactic fenestration, complications, and clinical variables were evaluated statistically.

**Results:**

Of 593 dogs undergoing cervical ventral slot surgery, 66.8% (396/593) received one or more prophylactic fenestrations. Fenestration was significantly more common in chondrodystrophic (CD) breeds (72.9%) compared to non-chondrodystrophic breeds (51.7%) (*p* < 0.001). The overall post-operative complication rate was 10.6% (63/593), with no significant association between complications and fenestration (*p* = 0.763), chondrodystrophy (*p* = 0.306) or site of herniation (*p* = 0.142).

**Conclusion:**

Prophylactic fenestration performed during cervical ventral slot surgery was not associated with an increased risk of perioperative complications. The significantly higher use of fenestration in chondrodystrophic dogs likely reflects their predisposition to early disc degeneration. These findings support the perioperative safety and potential effectiveness of fenestrations, particularly in dogs predisposed to disc degeneration. Prospective studies are warranted to evaluate its long-term benefits and biomechanical implications.

## Introduction

Intervertebral disc disease is among the most frequent canine neurological diseases in veterinary medicine, with a reported prevalence of 2% (1–4). Cervical Intervertebral Disc Herniation (IVDH) has been reported to represent between 12.9 and 25.3% of all patients suffering with IVDH (5, 6).

Intervertebral disc (IVD) degeneration is a key factor in the development of spinal disease in dogs (7). Hansen historically classified IVDH as type I, defined by disc extrusion secondary to chondroid degeneration, and type II, characterized by disc protrusion associated with fibrous degeneration (3, 8). These processes occur more frequently and at an earlier age in chondrodystrophic (CD) breeds, traditionally associated with Hansen type I herniations, while non-chondrodystrophic (NCD) breeds have been linked to type II herniations (7, 8). However, recent evidence suggests that both CD and NCD dogs exhibit similar histopathological changes, predominantly characterized by chondroid metaplasia (9). Clinical manifestations of cervical intervertebral disc herniation typically include cervical hyperaesthesia, ambulatory or non-ambulatory tetraparesis, nerve root signature, and, in severe cases, tetraplegia (7, 10–12).

Historically, treatment for IVDH II was performed with ventral fenestration of the IVD and removal of nuclear material through a fenestra on the annulus fibrosus (11, 13, 14). Fenestration alone at the cervical spine has been shown to be less effective than ventral slot for decompressive surgery and, following reports of complications and neurological deterioration, fenestration is currently questionable as a therapeutic option for cervical IVDH (3, 15, 16). Prophylactic fenestrations (PF) of the cervical intervertebral discs (IVDs) were recommended consistently for the anatomically first four cervical IVDs, with the addition of the next two if early signs of degeneration were observed (17), or alternatively for only the two discs immediately adjacent to the operated IVDs (11). Fenestration has been used for cervical disc prophylaxis and attempted for treating type II IVDH, but its limited success and potential to worsen dorsal annular protrusion have restricted its widespread adoption (7).

PF of the thoracolumbar intervertebral disc has been shown to significantly reduce the risks of future extrusion of the partially removed disc (18–20) and is now routinely performed (21). However, no consensus prevails regarding fenestration of cervical IVDs (3). Recurrence of symptoms following cervical IVDH was reported in 33 to 36% of dogs managed conservatively and in 5.3 to 19.6% of dogs that underwent surgical treatment for IVDH I (22–26). High rates of suspected cervical IVD re-extrusion, based on recurrence of clinical signs, highlight the need for research into methods that reduce the risk of further disc herniation. The standard ventral approach to cervical intervertebral disc extrusion provides access to the first six intervertebral discs. There is limited information available regarding the effects and risks of cervical fenestrations (24). Our objective was to determine the rate of complications in a large cohort of dogs undergoing cervical spinal cord decompression for IVDH I, with or without prophylactic fenestrations of degenerated discs, in a referral center where PF is routinely performed.

We hypothesize that prophylactic fenestration of cervical IVDs, when performed alongside decompressive surgery, does not increase the risk of peri-operative complications compared to standard surgery without fenestration.

## Materials and methods

### Study design and population

Dogs that underwent a ventral slot for an IVDH I, at a single referral institution between 2010 and 2020, were retrospectively identified from the hospital neurosurgical electronic database. Signed owner consent for the use of the clinical data was obtained on hospital admission for all of the patients. No ethical approval was obtained based on the strict retrospective nature of the study, the prior acquisition for use of patient data in clinical studies, and that no owner or referring veterinary surgeon was contacted. Complete clinical information for statistical analysis was required for inclusion.

A clinical neurological grade was determined, adapted from a previous described scoring system as described in Table 1 (27, 28).

**Table 1:** The neurological grading system adapted for use in the current study.

Grade	Description
0	Normal patients
1	Patients experiencing cervical hyperaesthesia with normal gait
2	Ambulatory tetraparesis, any degree of ataxia or lameness, with or without pain
3	Non ambulatory tetraparesis, with or without pain, ataxia, and/or lameness
4	Tetraplegia with intact deep pain nociception
5	Tetraplegia with absent deep pain nociception

The following data were extracted for each patient: date of presentation, signalment, neurological grade on admission; imaging findings, intervertebral disc space operated on and associated surgical procedure, any prophylactic fenestration was recorded as which space and the total number of intervertebral discs fenestrated. CD breeds were determined based on available studies (8, 18, 29–34).

Cases were excluded if the surgical procedure differed from a single ventral slot decompression, with or without prophylactic fenestrations, performed during a continuous anesthetic episode. Additional exclusions included incomplete medical records and imaging diagnoses other than Hansen Type I IVDH; cases with other diagnoses and complete records are detailed separately in the Appendix.

### MRI and surgical procedures

MRI images were acquired using a 0.4 tesla MRI scanner (Aperto Lucent, Hitachi Medical Corporation, Tokyo, Japan). All patients were scanned under general anesthesia positioned in dorsal recumbency. All images were reviewed and reported with a final diagnosis by a board-certified or ECVDI residency-trained radiologist. MRI sequences available for review, if required, included the following sequences: T2W sagittal, T2W transverse over the operated IVD. Imaging diagnosis was reported according to previously published data (30, 35). IVD degeneration has been determined based on T2W sagittal sequences. Although no formal grading was applied, degeneration was identified based on previously described MRI characteristics from the Pfirrmann classification, including loss of clear distinction between the nucleus pulposus and annulus fibrosus, nonhomogeneous disc structure, reduced signal intensity (intermediate to hypointense relative to CSF), decreased disc height, or complete disc space collapse (35, 36).

Surgical decompression targeted the identified compression site and was performed by an ECVN board-certified neurologist, or an ECVN residency trained neurologist. All dogs underwent ventral surgical decompression with a standard ventral slot (37).

Fenestration of degenerated intervertebral discs, determined according to MRI study, was performed via the same surgical approach, and involved excision of a rectangular section of the ventral annulus with an 11 blade, followed by partial removal of the underlying nucleus pulposus. When required, additional disc material was gently extracted using an arterial mosquito forceps.

### Immediate post-operative complications

Records were reviewed for any mention of complications in the immediate post-operative period defined as the period in hospital following surgery. Complications were characterized as a persistent pain, lack of expected neurological improvement, deterioration of the neurological status or death. When repeated imaging was available, consequent diagnosis was recorded.

### Statistical analysis

Statistical analyses were performed using Minitab21® and R 4.3.3.

Continuous and ordinal data are summarized by mean and standard deviation (SD) or by median and range, and binary and categorical data by frequencies and percentages. Comparison between categorical/binary variables was undertaken using chi-square tests of association, or by Fisher exact tests if counts were low. Ordinal and continuous data were compared between categories using Kruskal-Wallis tests, adjusted for ties. Ordinal and continuous data were compared to one another using Spearman rank correlation. In comparing the more common breeds a minimum threshold of 20 individuals/breed was applied. Significance was taken as *p* < 0.05.

## Results

### Case information

**Figure 1:**
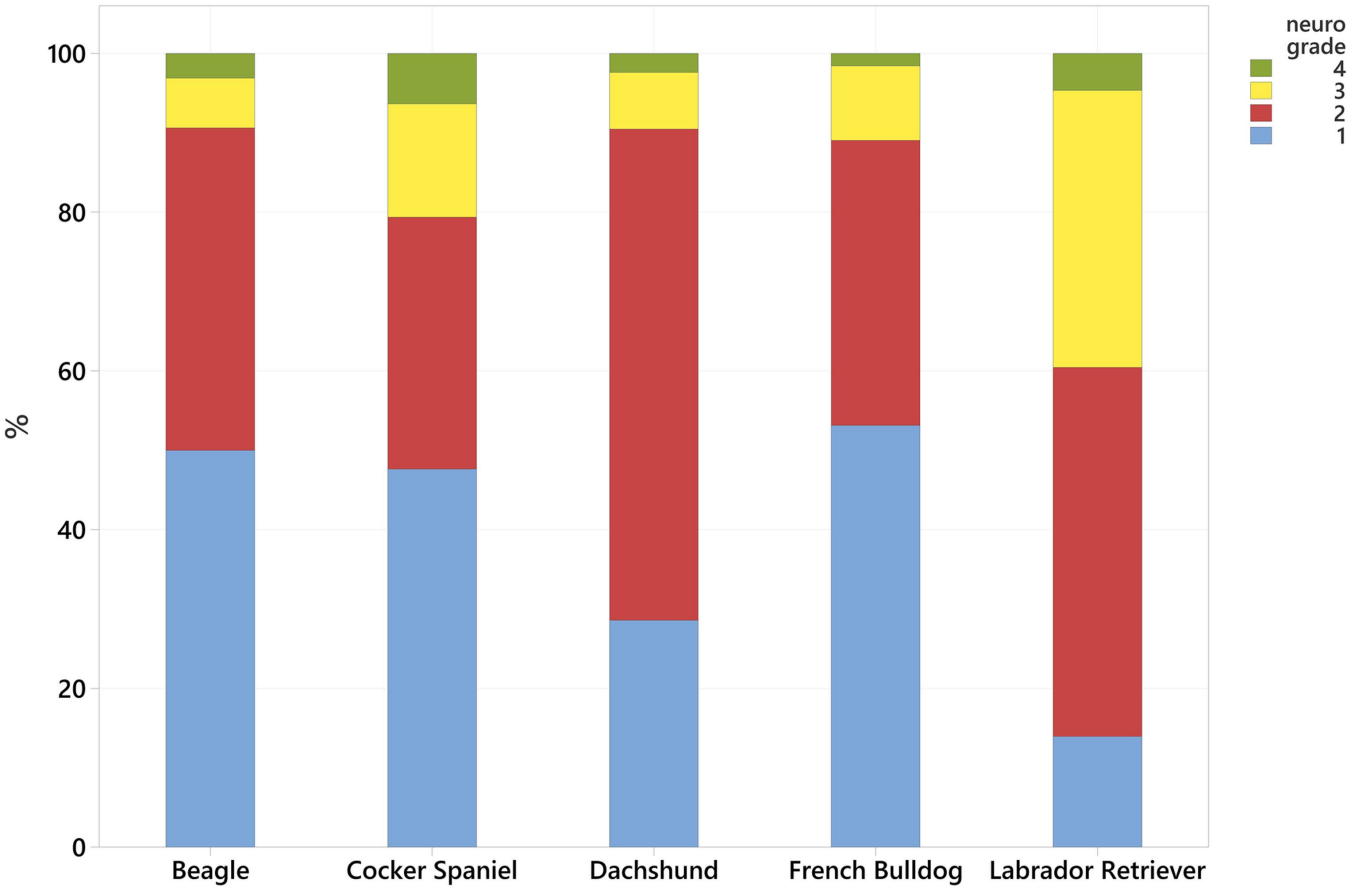
Neurological grade distribution (see Table 1, higher number more serious) for the most common breeds.

A total of 593 clients-owned IVDH I dogs undergoing a single ventral slot during the period reviewed were identified. Dogs of 71 Breeds were included. The most frequently affected breeds included French Bulldogs (128, 21.6%), Cocker Spaniels (63, 10.6%), Labrador Retrievers (43, 7.2%), crossbreeds (42, 7.1%), Dachshunds (42, 7.1%), Beagles (32, 5.4%), Cavalier King Charles Spaniels (17, 2.9%), Shih Tzus (13, 2.2%), Lhasa Apsos (12, 2.0%), Staffordshire Bull Terriers (12, 2.0%), Pugs (11, 1.8%), Springer Spaniels (11, 1.8%), Whippets (11, 1.8%) and Rottweilers (10, 1.7%). Fifty-seven breeds were presented with <10 dogs. Excluding crossbreeds, 402/551 dogs (73.0%) were CD. The mean (± SD) age of the studied population was 6.9 (± 2.7) years and ranged between 1.2 and 14.6 years. Sixty-two dogs (10.5%) were female, 179 (30.2%) were female spayed, 112 (18.9%) were male entire, and 240 (40.5%) male neutered. On examination, 220 (37.1%), had a neurological grade 1 on presentation. 262 (44.2%) with a grade 2, 96 (16.2%) with a grade 3 and 15 (2.5%) with a grade 4. There was a statistically significant difference in the distribution of neurological grades between the more common breeds (Kruskal-Wallis test *p* < 0.001); Labrador Retrievers having higher neurological grades on presentation compared to Beagles, Cocker Spaniels, Dachshunds and French Bulldogs (Figure 1).

The Spearman correlation between neurological grade and age was 0.245, indicating a weak positive correlation (*p* < 0.001), suggesting that higher neurological grades were associated with older dogs.

**Figure 2:**
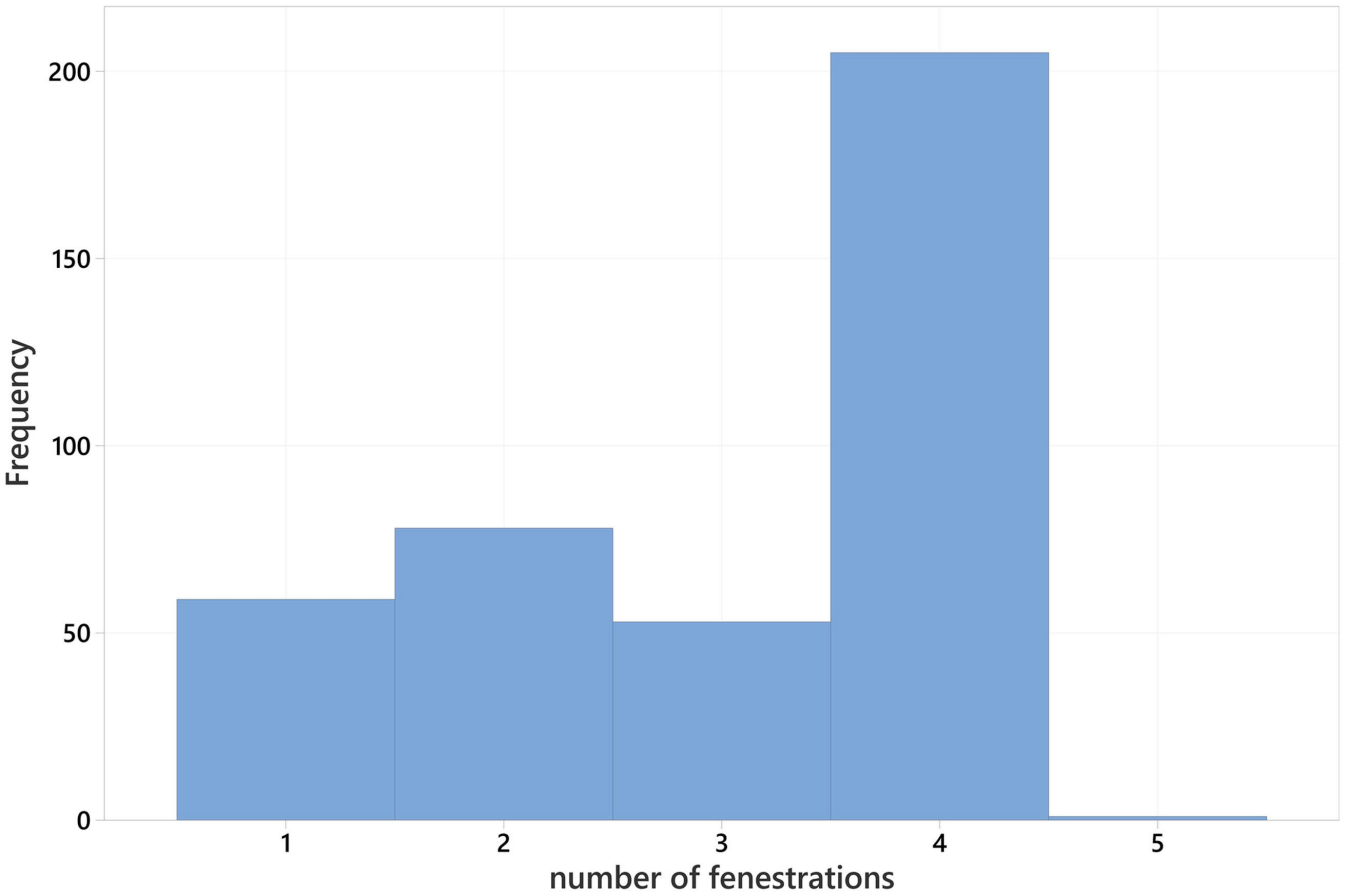
Number of prophylactic fenestrations (PF) performed per surgery, excluding non-fenestrations.

PFs were performed in 396 (66.8%) of the operated patients for a total of 1,199 fenestrations. Dogs received between one and five PFs, a mean of 3.03 ± 1.15 fenestrations were performed per case (Figure 2). Details about the distribution of fenestration sites relative to the operated intervertebral disc space are available in the Appendix.

No significant differences were identified in the proportion of dogs receiving PFs regarding sex (*p* = 0.351) or neurological grade (*p* = 0.812).

There was a significant difference in the proportion of fenestrations performed between the most common breeds (*p* < 0.001); most commonly occurring in French Bulldog (84.4%) and least often in Labrador Retriever (46.5%). Additionally, a significantly greater proportion of CD dogs received PFs compared to NCD dogs (72.9% vs. 51.7%, *p* < 0.001). At each neurological grade, CD dogs more frequently received fenestrations than NCD dogs [significant for grades 1 (*p* = 0.005), 2 (*p* = 0.007) and 4 (Fisher test *p* = 0.017), but not grade 3 (*p* = 0.057)].

The use of fenestration varied between the IVD operated on (*p* < 0.001); PF were more commonly performed during C3-C4 ventral slot (76.6%) and C4-C5 ventral slot (73.4%); and were less commonly performed during C7-T1 ventral slot (7.7%) (Table 2).

**Table 2:** Summary of study population by operated intervertebral disc (IVD) space with neurological grade, prophylactic fenestrations (PF), and complications.

Operated IVD space	Total cases (% of total)	Mean age (years)	Neurological grade distribution (%)				Total with PF (%)	Mean (SD) number of PFs	Total complications (%)
			Grade 1	Grade 2	Grade 3	Grade 4			
C2-C3	155 (26.1)	5.9	89 (57.4)	53 (34.2)	12 (7.7)	1 (0.6)	107 (69.0)	3.4 (1.0)	19 (12.3)
C3-C4	124 (20.9)	5.9	51 (41.1)	47 (37.9)	22 (17.7)	4 (3.2)	95 (76.6)	3.3 (1.0)	11 (8.9)
C4-C5	113 (19.1)	7.4	29 (25.7)	54 (47.8)	23 (20.3)	7 (6.2)	83 (73.4)	3.0 (1.1)	7 (6.2)
C5-C6	107 (18.0)	8.4	25 (23.3)	54 (50.5)	25 (23.4)	3 (2.8)	78 (72.9)	2.4 (1.2)	14 (13.1)
C6-C7	81 (13.7)	7.3	25 (30.9)	47 (58.0)	9 (11.1)	0	32 (39.5)	2.6 (1.3)	9 (11.1)
C7-T1	13 (2.2)	7.4	1 (7.7)	7 (53.8)	5 (38.5)	0	1 (7.7)	1 (−)	3 (23.1)
Total	593	7.1	220 (37.1)	262 (44.2)	96 (16.2)	15 (2.5)	396 (66.8)	3.0 (1.2)	63 (10.6)

### Complications

Sixty-three (10.6%) dogs suffered complications post-operatively, 48 (8.1%) dogs had repeated advanced MRI, followed by revision surgery, due to a neurological deterioration or lack of improvement. Persistent compression was the most frequent condition, with 28 cases (4.7%), of which two (0.3%) had a major haemorrhagic component. The second most frequent condition was surgical site infection (SSI) with 18 cases (3.0%). Finally, surgery on a wrong space occurred in two cases (0.3%), requiring revision surgery. Twelve dogs (2.0%) were humanely euthanised (PTS) while hospitalized following revision surgery. Fifteen dogs (2.5%) were PTS due to neurological deterioration following the initial surgery without repeating advanced imaging.

The proportion of complications diagnosed with repeated imaging did not differ significantly between dogs that did or did not have a PF (*p* = 0.736). Similarly, no significant association was observed between PF and dogs that were humanely euthanised (PTS) due to lack of improvement prior to hospital discharge, both in those without repeated MRI (*p* = 0.993) and when considering all dogs PTS in hospital, regardless of second imaging (*p* = 0.990). Overall complication rates, i.e., revision and/or PTS, did not differ significantly between PF (10.3%) and non-PF dogs (11.2%) (*p* = 0.763). No significant association was present between rate of complications and IVD operated on (*p* = 0.306), common breeds (Fisher test *p* = 0.447) or CD dogs (*p* = 0.142).

**Figure 3:**
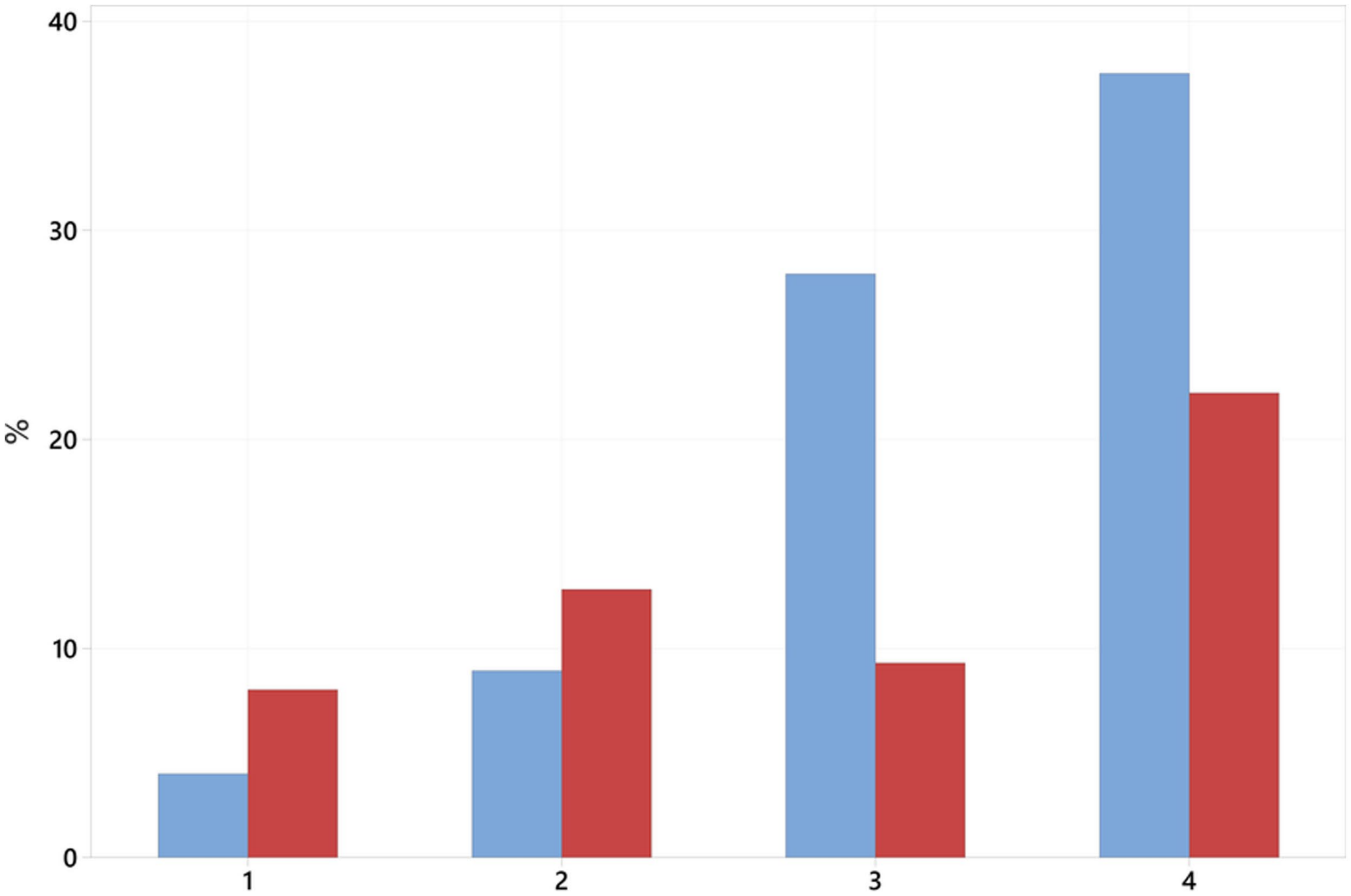
Percentage of complications (revisions + euthanasia) per neurological grade and prophylactic fenestration status. Blue bars represent no fenestration, red bars represent dogs receiving fenestration.

Overall complication rate did differ significantly between neurological grades (*p* = 0.007). Figure 3 illustrates the distribution of complications across neurological grades (1–4) and according to PF status.

## Discussion

This study presents a large retrospective analysis of 593 dogs undergoing ventral slot decompressive surgery. As previously reported, the C2-C3 IVD space was the most affected, and C7-T1 IVDH occurred less frequently overall (10, 16, 38, 39). French Bulldogs were the most frequently affected breed, but mixed-breed dogs constituted the largest group. A total of 66.8% of dogs received at least one prophylactic fenestration (PF). Of the more common breeds, French Bulldogs, had highest rates of fenestration and Labrador Retrievers the least. Labrador Retrievers presented with significantly higher neurological grades compared to other breeds, which may reflect breed-related clinical presentation, variations in owner decision-making, or referral patterns favoring surgical intervention in more severely affected individuals. Additionally, prophylactic fenestrations were significantly more common in CD breeds compared to NCD breeds, likely reflecting an increased predisposition to early disc degeneration in CD dogs. No other signalment or clinical variables showed significant differences between dogs receiving PF and those that did not.

The overall complication prevalence in our study population was approximately 10.6%, which is similar to the rate of 9.9% previously reported in a large retrospective study of adverse events following ventral slot surgeries in dogs; however, comparisons should be made cautiously, as a broader definition of adverse events was used, with major complications occurring in only 6.4% of cases (38). Euthanasia due to neurological deterioration occurred in 27 dogs (4.5%), of which 12 underwent revision surgery following repeated MRI confirmation of persistent spinal cord compression, while the remaining 15 were euthanised without repeated advanced imaging due to perceived lack of clinical improvement or further neurological deterioration. Our study overall euthanasia rate of 4.5% is higher than the 0.9% reported by Rossmeisl et al. (38). There was a significant link found between the neurological grade on presentation and overall complications. Sub-types of complications were defined by repeated advanced imaging or when euthanasia was directly performed due to a lack of expected response or to a deterioration. This may have underestimated the total number of cases where either imaging was not repeated, or euthanasia was not elected by the owners. In our study population, there was no significant association between fenestration and complications. Complications occurred more frequently following C7-T1 ventral slot, although based on small numbers and without statistical significance. This finding is compatible to what has been previously reported (38).

A study based on an online questionnaire addressed to veterinary surgeons reported a 45% rate of complications associated with fenestrations. The same study reported that only 25% routinely performed fenestrations on cervical IVDs prophylactic but the rate of complication per anatomical region operated was not specified. Our overall rate of complications is markedly lower, as in Suiter et al.’s study (25), where out of the 15 dogs receiving a ventral slot, only one suffered post-operative epidural hemorrhage leading to relapsing symptoms. In our study population, persistent spinal cord compression was the main reported diagnosis in 28 dogs undergoing a second MRI post-operatively, with 17/28 following a ventral slot either at C2-C3, C6-C7 or C7-T1. The second main reason reported for lack of expected outcome was surgical site infection in 18 dogs, equivalent to 3.0%. Nine of 18 episodes of infections occurred following a C2-C3 ventral slot. A lower rate of 1.3% was observed among 158 dogs undergoing spinal surgery, with surgical site infection; although those occurred exclusively in the thoracolumbar spine (40). Rossmeisl et al. reported the same rate of surgical site infection following ventral slots (38). Despite discospondylitis being described following fenestrations, those are complications usually seen after a lapse of time which was not in the scope of our study. Two cases involved surgery at the incorrect intervertebral space, and two experienced intraoperative hemorrhage, which corroborate previous suggestions as a rare complication. The latter is suspected secondary to injury to the veinous plexus (25, 38). In contrast to what has been reported in thoracolumbar fenestrations, the anatomy of the cervical spine implies that iatrogenic damage to vascular or nerve structures is less likely (3, 41).

The fact that the most extreme IVD are concerned with a higher complication rate can be secondary to the fact that exposure and visualization are more complex given the anatomy and surgical approach. Increased risks associated with the C7-T1 disc space have been reported, due to the complexity of surgical access and area visualization (38). Excision of the cranial bord of the manubrium and sternotomy have been described as a means to better expose IVDs from C6 to T2 and improve surgical decompression (38, 42, 43). No dogs were reported to have undergone a sternotomy in our study population and no statistical association to the IVD operated on was found.

Our findings indicate that prophylactic fenestrations were not associated with an increased risk of complications in the immediate post-operative period. In cases with repeated advanced imaging, no PF-specific complications were reported, and all observed complications were deemed secondary to the decompressive surgical procedure. Compared to ventral slot, fenestrations have been reported to be associated with less intraoperative complications (16). We believe that PF are uncomplicated surgical procedures compared to performing a ventral slot and possible complications are self-limiting and can be overcome with experience.

The significantly higher frequency of prophylactic fenestrations in CD dogs compared to NCD breeds likely reflects a clinical correlation with their predisposition toward earlier and more severe intervertebral disc degeneration. Specifically, this clinical correlation refers to the MRI identification of degenerative disc features, as fenestrations were primarily performed on discs showing degenerative changes on advanced imaging. CD breeds like French Bulldogs are predisposed to early IVD degeneration, with changes such as chondroid metaplasia occurring as early as 3–4 months and herniation developing by 3–7 years, mainly in the cervical and thoracolumbar spine. In contrast, NCD breeds develop degeneration later, around 6–8 years, characterized by gradual changes such as partial annulus rupture and degeneration of the nucleus pulposus, primarily in the caudal cervical and lumbosacral regions (3, 44). MRI effectively detects structural changes such as disc dehydration and herniation, aligning with histopathological observations (32).

The cervical spine functions as an interconnected biomechanical system, with higher mobility in the caudal segment compared to the cranial one (45, 46). The effects of prophylactic fenestration on spinal stability remain unclear. Ventral slot and fenestration may alter biomechanics and lead to instability (47). Macy et al. demonstrated increased sagittal motion at C5–C6 post-fenestration in healthy cadavers (48), potentially predisposing to adjacent segment disease. Discectomy has also been shown to induce rapid degenerative changes (49), and fenestration may trigger a progression from hypermobility to stiffness via spondylosis and fibrocartilaginous changes (48, 50, 51). These effects may vary between CD and NCD dogs, given the earlier and more severe degeneration in CD breeds (31, 52), though the overall biomechanical impacts of IVD degeneration in the cervical spine remains poorly understood (31). Multi-axial force interactions and degeneration severity are rarely quantified in clinical studies, and have not been a focus in thoracolumbar PF studies.

Concerns about IVD space collapse have been raised for both cervical and thoracolumbar fenestration (7), particularly in the caudal cervical spine, where mobility is higher and associated with cervical spondylomyelopathy (CSM). While instability is a suspected factor in CSM, recent evidence suggests a possible genetic basis in young dogs (46, 53). Instability has also been linked to IVD disease in small breeds (54), but outcomes do not appear to differ significantly between decompression with or without stabilization (55). Stabilization itself may affect adjacent discs, potentially leading to degeneration, and cadaver models have limitations due to the absence of soft tissues and dynamic loading (56). Assessment of instability is challenging, particularly intraoperatively with a predicted high variability according to the breed (54).

The retrospective nature of our study introduced challenges such as non-standardized clinical assessments, variability in treatment protocols, inconsistent record-keeping, and differences in surgeons’ experience. While extensive clinical data were available for reviewing, some perioperative factors such as the degree of disc degeneration, were rarely documented and could not be included in the study. Assessing individual imaging studies to determine how degeneration severity influenced surgical planning was beyond the scope of this study.

Our study focused on major post-operative complications, defined as a lack of expected improvement following surgery. We did not analyze intra-operative concerns that were resolved without leading to post-operative deterioration or delayed recovery. Fenestrations have been reported to increase the risk of intra-operative hemorrhage and prolong surgical time. As these factors were not strictly monitored in our study, they may have been underreported. However, we hypothesize that such events would likely have a significant impact only if they resulted in delayed recovery. Nevertheless, prolonged surgical time or hospital stay could have financial implications, which were not assessed in our study population. However, our primary objective was to study fenestrations performed in the context of an initial surgical intervention and to compare the associated risks.

In the absence of an observed increase in post-operative complications in our study population, PF may confer clinical benefit, particularly in CD dogs affected with IVD degeneration and at risk for future IVDH recurrence. As PF is likely to prolong surgical time, future studies should control for this variable when evaluating its overall risk–benefit profile, even though no increase in complications was identified. Equally, the biomechanical consequences of PF remain poorly understood. The limited availability of advanced modeling techniques, such as finite element analysis, constrains our ability to assess the long-term mechanical impact of fenestration and its potential role in secondary pathology. Future studies should incorporate standardized criteria for fenestration and disc degeneration, alongside biomechanical and follow-up investigations, to better inform surgical decision-making and improve outcomes in dogs with cervical IVDH.

## Conclusion

This retrospective study demonstrates that prophylactic cervical disc fenestration, as performed in conjunction with ventral slot decompression, was not associated with an increased rate of post-operative complications. The high frequency of fenestration in chondrodystrophic dogs likely reflects a clinical correlation with early MRI-visible disc degeneration. Although variation in surgical decision-making and documentation limited evaluation of degeneration severity, our findings suggest that prophylactic fenestration may represent a safe adjunct to decompression surgery, particularly in breeds predisposed to early-onset intervertebral disc disease. Given the current uncertainty surrounding the biomechanical effects of fenestration, especially in non-degenerated discs, further research is warranted. Future studies should incorporate standardized imaging-based criteria for disc degeneration and investigate the long-term outcomes and mechanical implications of fenestration to better guide surgical practice in cervical IVDH.

## Data Availability

The raw data supporting the conclusions of this article will be made available by the authors, without undue reservation.
