# Supplementary material for: Post-operative complications following cervical ventral slot in dogs: a retrospective review of the influence of prophylactic fenestration and chondrodystrophy in 593 cases
Source: Front Vet Sci. 2025 Jul 29;12:1616461. doi: 10.3389/fvets.2025.1616461 (PMC12341391; doi:10.3389/fvets.2025.1616461)
Supplement: Supplementary file 1 [file Table_1.docx]

Supplementary Material

**Appendix.** Summary of other disease types, with IVDH I included for reference

| **Disease** | **Total cases  (% of total)** | **Mean age (years)** | **Chondrodystrophic (%)** | **Neurological Grade Distribution (%)** | | | | **Total with PF  (%)** | **Mean (SD) number of PFs** | **Total complications (%)** |
| --- | --- | --- | --- | --- | --- | --- | --- | --- | --- | --- |
|  |  |  |  | Grade 1 | Grade 2 | Grade 3 | Grade 4 |  |  |  |
| Discospondylitis | 2 (0.3) | 5.6 | 0 | 1 (50.0)) | 1 (50.0) | 0 | 0 | 1 (50.0) | 1.0 (-) | 0 |
| HNPE | 54 (7.7) | 9.4 | 24 (51.0) | 3 (5.6) | 17 (31.5) | 26 (48.1) | 8 (14.8) | 37 (68.5) | 2.6 (1.3) | 7 (13.0) |
| IVDH II | 50 (7.2) | 7.1 | 22 (44.9) | 13 (26.0) | 27 (54.0) | 7 (14.0) | 3 (6.0) | 27 (54.0) | 2.7 (1.4) | 8 (16.0) |
| **IVDH 1** | 593 (84.8) | 6.9 | 402 (73.0) | 220 (37.1) | 262 (44.2) | 96 (16.2) | 15 (2.5) | 396 (66.8) | 3.0 (1.2) | 63 (10.6) |
| Total | 699 | 7.1 | 448 (69.1) | 237 (33.9) | 307 (43.9) | 129 (18.4) | 26 (3.7) | 461 (65.1) | 3.0 (1.2) | 78 (11.2) |
