# Supplementary material for: Post-operative complications following cervical ventral slot in dogs: a retrospective review of the influence of prophylactic fenestration and chondrodystrophy in 593 cases
Source: Front Vet Sci. 2025 Jul 29;12:1616461. doi: 10.3389/fvets.2025.1616461 (PMC12341391; doi:10.3389/fvets.2025.1616461)
Supplement: Supplementary file 2 [file Table_2.docx]

| **Operated IVD space** | **Dogs with PF (n)** | **Number cases per total PF/surgery (1–4)** | | | | **Fenestrated site**  **Count of IVD with PF (% n)** | | | | | |
| --- | --- | --- | --- | --- | --- | --- | --- | --- | --- | --- | --- |
|  |  | **1** | **2** | **3** | **4** | **C2-C3** | **C3-C4** | **C4-C5** | **C5-C6** | **C6-C7** | **C7-T1** |
| C2–C3 | 107 | 10 | 10 | 12 | 75 | X | 98 (91.6%) | 92 (86.0%) | 92 (86.0%) | 84 (79.1%) | 0 |
| C3–C4 | 95 | 9 | 10 | 19 | 57 | 78 (81.0%) | X | 41 (43.2%) | 81 (85.3%) | 71 (74.7%) | 0 |
| C4–C5 | 83 | 9 | 25 | 10 | 39 | 48 (57.8%) | 65 (78.3%) | X | 76 (91.6%) | 29 (34.9%) | 0 |
| C5–C6 | 78 | 22 | 22 | 11 | 23 | 31 (39.7%) | 32 (41.0%) | 40 (51.3%) | X | 67 (85.9%) | 0 |
| C6–C7 | 32 | 8 | 9 | 3 | 12 | 12 (37.5%) | 15 (46.9%) | 21 (65.6%) | 21 (65.6%) | X | 5 (15.6%) |
| C7-T1 | 1 | 1 | 0 | 0 | 0 | 0 | 0 | 0 | 0 | 1  (100%) | X |

**Appendix Table 2.** Distribution of prophylactic fenestrations (PFs) by operated intervertebral disc (IVD) space: number of dogs, number of PFs performed per case, and percentage of dogs receiving PF at each disc site.
